# Adomian decomposition sumudu transform method for solving a solid and porous fin with temperature dependent internal heat generation

**DOI:** 10.1186/s40064-016-2106-8

**Published:** 2016-04-19

**Authors:** Trushit Patel, Ramakanta Meher

**Affiliations:** Department of Applied Mathematics and Humanities, SVNIT, Icchanath Circle, Surat, 395007 India

**Keywords:** Adomian decomposition sumudu transform method, Fractional order differential equation, Caputo fractional derivative, Porous fin, Temperature dependent thermal conductivity, Internal heat generation, Thermal analysis

## Abstract

In this paper, Adomian decomposition sumudu transform method is introduced and used to solve the temperature distribution in a solid and porous fin with the temperature dependent internal heat generation for a fractional order energy balance equation. In this study, we assume heat generation as a variable of fin temperature for solid and porous fin and the heat transfer through porous media is simulated by using Darcy’s model. The results are presented for the temperature distribution for the range of values of parameters appeared in the mathematical formulation and also compared with numerical solutions in order to verify the accuracy of the proposed method. It is found that the proposed method is in good agreement with direct numerical solution.

## Background

Fins are commonly used to facilitate the dissipation of heat from a heated wall to the surrounding environment. Examples of fin are the radiator in vehicles and heat exchangers in power plants. In electrical devices like motors and transformers, the generated heat can be efficiently transferred. In the study of heat transfer, fin is a surface, made by metallic material which is used to increase the rate of heat transfer to the environment. The rate of heat transfer depends on the surface area of the fin. Fins are extensively used to improve the rate of heat dissipation from a hot surface, especially in thermal engineering applications (Bergman et al.; Nield and Bejan).

In many thermal engineering applications, convective flow through porous media is mandatory for the investigation. Several numerical and analytical revisions has been conducted so far to afford a profound understanding of the transport system of the heat transfer inside the porous medium. Generally, high thermal conductivity porous substrates are employed to improve the rate of forced convection heat transfer in many engineering applications such as reactor heat exchangers, solar collectors and in cooling process (Alkam and Al-Nimr 1999). However, heat transfer in porous fins has attracted a lot of attention of researchers with a wide range of it’s applications, especially in recent years. Kiwan and Al-Nimr (2001) was the first person who introduced the concept of fins made of porous materials by introducing Darcy’s model (Kiwan 2007; Kiwan and Zeitoun 2008).

Now a days, Heat exchanger industries are looking for more compact and cost–effective heat exchanger manufacturing techniques which leads to use porous fins in enhance heat transfer (Kiwan 2007). The heat-transfer enhancement between two parallel-plate channels was investigated by adding porous fin through the channel (Hamdan and Moh’d 2010) and by adding porous insert to one side of the duct walls (Hamdan et al. 2000). Alkam et al. (2002) investigated the thermal analysis of natural convection porous fins. They studied all the geometric flow parameters that influence the temperature distribution in to a single parameter specified *S*_*h*_. They considered three cases: the infinite fin, a finite fin with an insulated tip and a finite fin with uninsulated tip. Similarly Gorla and Bakier (2011) discussed the thermal analysis of natural convection and radiation in the porous fin and showed that the radiation transfers more heat than a similar model without radiation. Hatami et al. (2013) studied the heat transfer through porous fin with different porous material and compared their results with the Differential Transform Method, Collocation Method and Least Square Method. They Hatami and Ganji (2013) also studied the thermal performance of circular convective–radiative porous fins with different section, shapes and materials. Ghasemi et al. (2014) used the Differential Transform Method for solving the nonlinear temperature distribution in solid and porous fin with temperature dependent internal heat generation. Patel and Meher (2015a, b) studied the fractional solution of longitudinal porous fin for the case of temperature distribution, efficiency and effectiveness and also analysed the variation of temperature distribution for a straight rectangular fin with power-law temperature dependent surface heat flux by using Adomian decomposition sumudu transform method.

It is revealed that, the concept of fractional derivative is more suitable for modeling real world problem than the local derivative. Many researchers have devoted their attention in developing new definition of fractional derivative (Atangana 2016). Baskonus and Bulut (2015) applied the fractional Adams–Bashforth–Moulton Method for obtaining the numerical solutions of some linear and nonlinear fractional ordinary differential equations. Baskonus and Bulut (2015) studied it to obtain some new analytical solutions to the (1 + 1)-dimensional nonlinear Dispersive Modified Benjamin Bona Mahony equation by using modified exp-function method. Roshid et al. (2014) studied solitary wave solutions for vakhnenko-parkes equation via exp-function and Exp $(-\phi (\xi ))$—expansion method. Also they Roshid et al. (2014) studied traveling wave solutions of nonlinear partial differential equation via new extended $(G^{\prime }/G)$—expansion method.

In the present study, we fractionalize the energy balance equation in order to understand the anomalous behavior of this system and to find the temperature distribution in solid and porous fin by using Adomian decomposition sumudu transform method.

## Preliminaries

### Definitions of Caputo fractional derivative

In this part of the paper it would be useful to introduce some definitions and properties of the fractional calculus theory. There are several definitions of fractional derivatives of order $\alpha >0$ (Miller and Ross; Srivastava et al. 2014). The two most commonly used definitions are Riemann-Liouville and Caputo.

#### **Definition**

The Riemann-Liouville fractional integral of fractional order is defined as Miller and Ross

1 $$J^{\alpha } f(t)=\frac{1}{\Gamma (\alpha )} \int _{0}^{t}(t-\tau )^{\alpha -1} f(\tau )d\tau ,\quad \alpha \in R^{+}$$

where $R^{+}$ is the set of positive real numbers and $\Gamma (\cdot )$ is the gamma function.

#### **Definition**

The fractional derivative of *f*(*t*) in the Caputo sense is defined by

2 $$\begin{aligned} D_t^\alpha f(t)&= {J^{m - \alpha }}{D^m}f(t) \\&= \left\{ \begin{array}{ll} \frac{1}{{\Gamma (m - \alpha )}}\;\int \nolimits _0^t {{(t - \tau )}^{(m - \alpha - 1)}}\frac{{{d^m}f(\tau )}}{{d{\tau ^m}}},&\quad {if}\;\;m - 1< \alpha < m,\;m \in N \\ \frac{{{d^m}f(t)}}{{d{t^m}}}&\quad {if} \;\; \alpha = m,\ m \in N \end{array} \right. \end{aligned}$$

where the parameter *α* is the order of the derivative and is allowed to be real or even complex. Here *N* is the set of natural numbers. In this paper only real and positive *α* will be considered. The properties underpinning the use of the Caputo derivative can be found in Atangana (2016), Atangana and Alqahtani (2016) and Atangana and Baleanu (2016).

### Basics of sumudu transform method

The sumudu transform is a new integral transform (Kadem and Baleanu 2012; Atangana and Baleanu 2013; Jarad et al. 2012) which is a little known and not widely used whose defined for the functions of exponential order.

#### **Definition**

The sumudu transform of a function *f*(*t*), defined for all real numbers $t \ge 0$, is the function *F*(*u*), defined by Watugala (1993)

3 $$F(u) = S[f(t)] = \int _0^\infty {\frac{1}{u}f} (t)\,{e^{ - \left( {\frac{t}{u}} \right) }}dt,\quad u \in ( - {\tau _1},\;{\tau _2})$$

#### **Definition**

Sumudu transform of function derivatives is defined as Belgacem and Karaballi (2006)

4 $$S[f^{(n)} (t)]=\frac{S\left[ f(t)\right] }{u^{n} } -\sum _{k=0}^{n-1}\frac{f^{(k)} (0)}{u^{n-k} }$$

#### **Definition**

Sumudu transform of the Caputo fractional derivative is defined as Belgacem and Karaballi (2006)

5 $$\begin{aligned} S\left[ D_{t}^{\alpha } f(t)\right] =u^{-\alpha } S[f(t)]-\sum _{k=0}^{m-1}u^{-\alpha +k} f^{(k)} (0+) ,\quad m-1<\alpha \le m \end{aligned}$$

#### **Theorem**

*LetG*(*u*) *be the sumudu transform off*(*t*) *such that*

1. $\left( G(1/s)/s\right)$*is a meromorphic function, with singularities having*${\mathrm{Re}} [s] \le \gamma ;$
2. *There exist a circular region*$\gamma$*with radiusRand positive constantsMandKwith*$\left| {G(1/s)/s} \right| < M{R^{ - K}}$, *then the functionf*(*t*) *is given by*

6 $$\begin{aligned} {S^{-1}}[F(s)]&= \frac{1}{{2\pi i}}\int \limits _{\gamma - i\infty }^{\gamma + i\infty } {{e^{st}}F\left( {\frac{1}{s}} \right) \frac{{ds}}{s}} \\&= \sum {resdidues\;\left[ {\frac{{{e^{st}}F\left( {\frac{1}{s}} \right) }}{s}} \right] } \end{aligned}$$

For the proof see Belgacem and Karaballi (2006).

## Formulation of Adomian decomposition sumudu transform method (ADSTM)

Consider a general fractional nonlinear nonhomogeneous differential equation with satisfying the initial condition of the form, as,

7 $$\frac{d^{\alpha } \theta (\zeta )}{d\zeta ^{\alpha } } +R\theta (\zeta )+N\theta (\zeta )=g(\zeta )$$

subject to the initial condition

8 $$\theta (0)= K$$

where $\frac{d^{\alpha } }{d\zeta ^{\alpha } }$ denotes without loss of generality the Caputo fraction derivative operator, *R* is the linear differential operator, *N* represents the nonlinear differential operator, and $g(\zeta )$ is the source term.

On applying the sumudu transform and the Caputo fractional derivative in Eq. (7), it obtains

9 $$S[\theta (\zeta )]=\theta (0)+u^{\alpha } S[g(\zeta )]-u^{\alpha } S[R\theta (\zeta )+N\theta (\zeta )]$$

Sumudu inverse transform of Eq. (9), gives

10 $$\theta (\zeta )= K + S^{-1} \left[ u^{\alpha } S[ g(\zeta ) - R\theta (\zeta ) - N \theta (\zeta )]\right]$$

where $\theta (0) = K$ is prescribed initial conditions.

Using Adomian decomposition method which obtains the approximate solution of Eq. (10) in a series form as Adomian

11 $$\theta (\zeta )=\sum _{n=0}^{\infty }\lambda ^{n} \theta _{n} (\zeta )$$

and the nonlinear term can be expressed as a sum of Adomian Polynomials

12 $$N\theta (\zeta )=\sum _{n=0}^{\infty }\lambda ^{n} A_{n} (\theta (\zeta ))$$

where the Adomian polynomials $A_{n} (\theta )$, depends upon the solution components $\theta _{0} , \theta _{1} , \theta _{2},\ldots , \theta _{n}$, can be defined as follows

13 $$\begin{aligned} A_{n} (\theta _{0} , \theta _{1} , \theta _{2} ,\ldots , \theta _{n} )=\frac{1}{n!} \frac{\partial ^{n} }{\partial \lambda ^{n} } \left[ N\left( \sum _{i=0}^{\infty }\lambda ^{i} \theta _{i} \right) \right] _{\lambda =0} , \quad for \ \ n= 0, 1, 2, \ldots \end{aligned}$$

On substituting Eqs. (11) and (12) in Eq. (10), it obtains

14 $$\begin{aligned} \sum _{n=0}^{\infty }\lambda ^{n} \theta _{n} (\zeta ) = K + \lambda \left[ S^{-1} \left[ u^{\alpha } S\left[ g(\zeta ) - R\sum _{n=0}^{\infty }\lambda ^{n} \theta _{n} (\zeta ) - \sum _{n=0}^{\infty }\lambda ^{n} A_{n} (\theta ) \right] \right] \right] \end{aligned}$$

The resulting Eq. (14) is the coupling of the Adomian decomposition method and the sumudu transform. On comparing the coefficients of like powers of $\lambda$, the recursive relation of Eq  (14) can be written as

15 $$\begin{aligned} \theta _{0} (\zeta )&= K \\ \theta _{1} (\zeta )&= S^{-1} \left[ u^{\alpha } S\left[ g(\zeta ) - R\theta _{0} (\zeta )- A_{0} (\zeta )\right] \right] \\ \theta _{n+1} (\zeta )&= - S^{-1} \left[ u^{\alpha } S\left[ R\theta _{n} (\zeta )+A_{n} (\zeta )\right] \right] \quad n = 1,2,3,\ldots \end{aligned}$$

Since $\sum _{n=0}^{\infty } \theta _{n} (\zeta )$ is a rapidly converging series, the partial sum $\phi _{m}=\sum _{i=0}^{m-1} \theta _{i} (\zeta )$ is our approximant to the solution (Adomian).

## Problem description

Here we considered two cases, namely (1) solid fin and (2) porous fin to study the fin temperature distribution in longitudinal fin with rectangular profile.

### Case 1: solid fin with temperature dependent internal heat generation and constant thermal conductivity

**Fig. 1:**
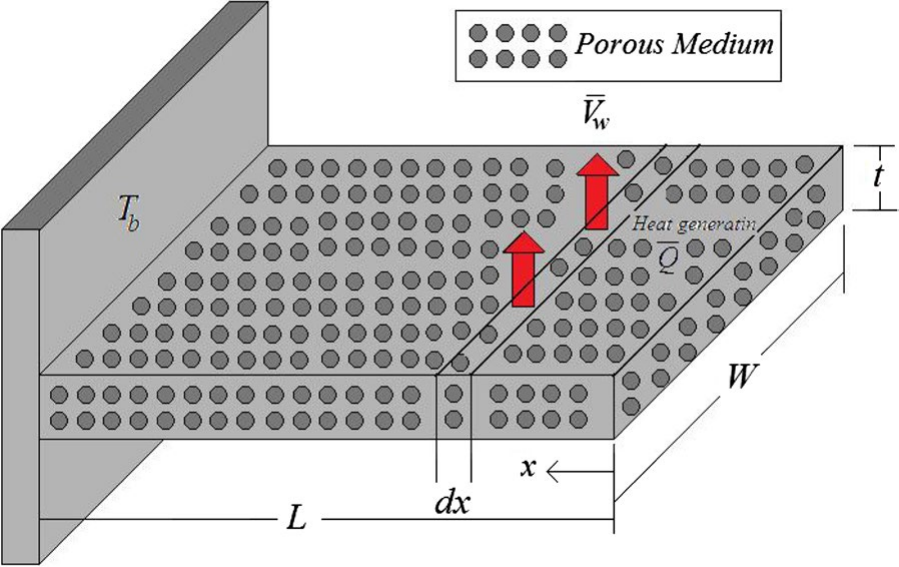
Schematic of fin geometry with the internal heat generation source

Consider a longitudinal fin with a constant rectangular profile, section area *A*, length *L*, perimeter *P*, thermal conductivity *k*, and heat generation $\bar{Q}$. Fin is attached to a surface with constant temperature $T_{b}$ and losses heat to the surrounding medium with temperature $T_{\infty }$ through a constant convective heat transfer coefficient *h*. Here we assumed that the temperature variation in the transfer direction is negligible, so heat conduction occurs only in the longitudinal direction (*x* direction). A schematic diagram of the described fin is shown in Fig. 1.

The governing differential equation and boundary condition for this problem can be written as Ghasemi et al. (2014)

16 $$\frac{d^{2} T}{dx^{2} } -\frac{hP}{kA} \left( T-T_{\infty } \right) +\frac{\bar{Q}}{k} =0$$

17 $$\left[ \frac{dT}{dx} \right] _{x=0} =0$$

18 $$\left[ T\right] _{x=L} =T_{b}$$

Here it is assumed that the temperature heat generation in the solid fin varies with temperature $T_{\infty }$,that can be defined as

19 $$\bar{Q}=\bar{Q}_{\infty } (1+I(T-T_{\infty } ))$$

Where $\bar{Q}_{\infty }$ is the internal heat generation at temperature $T_{\infty }$. On introducing the dimensionless variables

20 $$\begin{aligned} \theta&= \frac{(T-T_{\infty } )}{(T_{b} -T_{\infty } )} ,\quad \zeta =\frac{x}{L} ,\quad M^{2} =\frac{hPL^{2} }{k_{0} A} , \\ G&= \frac{\bar{Q}_{\infty } }{hP(T_{b} -T_{\infty } )}, \quad I_{G} = I(T_{b} -T_{\infty } ) \end{aligned}$$

The dimensionless form of Eqs. (16)–(18) can be written as

21 $$\frac{d^{2} \theta }{d\zeta ^{2} } -M^{2} \theta +M^{2} G(1+I_{G} \theta )=0$$

22 $$\zeta =0,\quad \frac{d\theta }{d\zeta } =0$$

23 $$\zeta =1,\quad \theta =1$$

To understand the anomalous behavior of this system, we fractionalize the energy balance Eq. (21) into fractional order ($\alpha >0$) in order to find the fin temperature in solid fins as,

24 $$\frac{d^{\alpha } \theta }{d\zeta ^{\alpha } } - M^{2} \theta +M^{2} G(1+I_{G} \theta )=0,\quad 1<\alpha \le 2\; and\; 0\le \zeta \le 1$$

With boundary conditions

25 $$\left. \frac{d\theta }{d\zeta } \right| _{\zeta =0} =0 \quad and \quad \left. \theta \right| _{\zeta =1} =1.$$

Now, applying sumudu transform on both sides of Eq. (24), we obtain

26 $$S[\theta (\zeta )] = K + u^{\alpha }S\left[ (M^{2} - M^{2}G I_{G}) \theta - M^{2}G\right]$$

taking inverse sumudu transform on both side of Eq. (26), we get

27 $$\theta (\zeta ) = K + S^{-1} \left[ u^{\alpha }S\left[ (M^{2} - M^{2}G I_{G}) \theta - M^{2}G\right] \right]$$

Now on applying Adomian decomposition method it obtains,

28 $$\begin{aligned} \sum _{n=0}^{\infty }\lambda ^{n} \theta _{n} (\zeta ) = K + \lambda \left[ S^{-1} \left[ u^{\alpha } S\left[ \sum _{n=0}^{\infty }\lambda ^{n}(M^{2} - M^{2}G I_{G}) \theta _{n}(\zeta ) - M^{2}G \right] \right] \right] \end{aligned}$$

Comparing the coefficients of $\lambda$ in Eq. (28), we have

$$\begin{aligned} \theta _{0} \left( \zeta \right)&= K \\ \theta _{1} \left( \zeta \right)&= S^{-1} \left[ u^{\alpha } S\left[ \theta _{0} \left( \zeta \right) \left( M^{2} -M^{2} GI_{G} \right) - M^{2}\theta _{0}(\zeta )\right] \right] \\&= \frac{KM^{2} (1-GI_{G} )\, \zeta ^{\alpha } }{\Gamma (\alpha +1)} -\frac{M^{2} G\, \zeta ^{\alpha } }{\Gamma (\alpha +1)} \\ \theta _{2} \left( \zeta \right)&= S^{-1} \left[ u^{\alpha } S\left[ \theta _{1} \left( \zeta \right) \left( M^{2} -M^{2} GI_{G} \right) \right] \right] \\&= \frac{KM^{4} (1-GI_{G} )^{2} \zeta ^{2\alpha } }{\Gamma (2\alpha +1)} -\frac{M^{4} (1-GI_{G} )G\zeta ^{2\alpha } }{\Gamma (2\alpha +1)} \\ \theta _{3} \left( \zeta \right)&= S^{-1} \left[ u^{\alpha } S\left[ \theta _{2} \left( \zeta \right) \left( M^{2} -M^{2} GI_{G} \right) \right] \right] \\&= \frac{KM^{6} (1-GI_{G} )^{3} \zeta ^{3\alpha } }{\Gamma (3\alpha +1)} -\frac{M^{6} (1-GI_{G} )^{2} G\zeta ^{3\alpha } }{\Gamma (3\alpha +1)} \\ \theta _{4} \left( \zeta \right)&= S^{-1} \left[ u^{\alpha } S\left[ \theta _{3} \left( \zeta \right) \left( M^{2} -M^{2} GI_{G} \right) \right] \right] \\&= \frac{KM^{8} (1-GI_{G} )^{4} \zeta ^{4\alpha } }{\Gamma (4\alpha +1)} -\frac{M^{8} (1-GI_{G} )^{3} G\zeta ^{4\alpha } }{\Gamma (4\alpha +1)} \\ \vdots&\;&\end{aligned}$$

Summing these terms, the final temperature field $\theta \left( \zeta \right)$, is calculated up to five terms as follows

29 $$\begin{aligned} \theta \left( \zeta \right)& \cong K-\frac{M^{2} G\, \zeta ^{\alpha } }{\Gamma (\alpha +1)} +\frac{KM^{2} (1-GI_{G} )\, \zeta ^{\alpha } }{\Gamma (\alpha +1)} -\frac{M^{4} (1-GI_{G} )G\zeta ^{2\alpha } }{\Gamma (2\alpha +1)} \\&\quad + \frac{KM^{4} (1-GI_{G} )^{2} \zeta ^{2\alpha } }{\Gamma (2\alpha +1)} - \frac{M^{6} (1-GI_{G} )^{2} G\zeta ^{3\alpha } }{\Gamma (3\alpha +1)} +\frac{KM^{6} (1-GI_{G} )^{3} \zeta ^{3\alpha } }{\Gamma (3\alpha +1)} \\&\quad -\frac{M^{8} (1-GI_{G} )^{3} G\zeta ^{4\alpha } }{\Gamma (4\alpha +1)} + \frac{KM^{8} (1-GI_{G} )^{4} \zeta ^{4\alpha } }{\Gamma (4\alpha +1)} \end{aligned}$$

Equation (29) represents the expression for finding the temperature distribution in solid fin of fractional order energy balance Eq. (24), where the coefficient *K* denotes the fin tip temperature, and it can be determined at the boundary condition $\left. \theta \right| _{\zeta =1} =1$.

### Case 2: porous fin with the temperature dependent internal heat generation

Here the energy balance equation for rectangular porous profile fin with temperature dependent internal heat generation can be written as

30 $$\begin{aligned} Q(x)-Q(x+\Delta x)+\bar{Q}\cdot A\, \cdot \Delta x & =\bar{m}\cdot c_{p} \left[ T(x)-T_{\infty } \right]\\&\quad +h(p\cdot \Delta x)\left[ T(x)-T_{\infty } \right] \end{aligned}$$

The mass flow rate of the fluid passing through the porous material can be written as

31 $$\bar{m}=\rho \cdot \bar{V}_{w} \cdot \Delta x\cdot w$$

From Darcy’s model, the passage velocity (Hatami et al. 2013) is

32 $$\bar{V}_{w} =\frac{g\cdot k\cdot \beta \cdot (T(x)-T_{\infty } )}{v}$$

On substituting Eqs. (31) and (32) into Eq. (30), it gives

33 $$\begin{aligned} \frac{Q(x)-Q(x+\Delta x)}{\Delta x} + \bar{Q}\cdot A&= \frac{\rho \cdot g\cdot k\cdot \beta \cdot \left( T(x)-T_{\infty } \right) ^{2} \cdot w\cdot c_{p} }{v} \\&\quad + h\cdot p\left( T(x)-T_{\infty } \right) \end{aligned}$$

As $\Delta x\rightarrow 0$, Eq. (33) becomes

34 $$\begin{aligned} \frac{dQ}{dx} +\bar{Q}\cdot A =\frac{\rho \cdot g\cdot k\cdot \beta \cdot \left( T(x)-T_{\infty } \right) ^{2} \cdot w\cdot c_{p} }{v} +h\cdot p\left( T(x)-T_{\infty } \right) \end{aligned}$$

Also, from Fourier’s law of conduction

35 $$Q=-k_{eff} A\frac{dT}{dx}$$

where $k_{eff}$ is the effective thermal conductivity of the porous fin, that can be obtained from the following equation (Hatami et al. 2013)

36 $$k_{eff} =\psi k_{f} +(1-\psi )k_{s}$$

Where $\psi$ is the porosity of the porous fin. On substituting Eq. (35) into Eq. (34), it gives

37 $$\begin{aligned} \frac{d^{2} T}{dx^{2} } +\frac{\bar{Q}}{k_{eff} } =\frac{\rho \cdot g\cdot k\cdot \beta \cdot \left( T(x)-T_{\infty } \right) ^{2} \cdot w\cdot c_{p} }{v} +\frac{h\cdot p}{k_{eff} A} \left( T(x)-T_{\infty } \right) \end{aligned}$$

On introducing the dimensionless variables and numbers

38 $$\begin{aligned} \theta&= \frac{(T-T_{\infty } )}{(T_{b} -T_{\infty } )} ,\quad \zeta = \frac{x}{L} ,\, \, M^{2} = \frac{hPL^{2} }{k_{0} A} ,\quad G = \frac{\bar{Q}_{\infty } }{hP(T_{b} -T_{\infty } )} , \\ I_{G}&= I(T_{b} - T_{\infty } ),\quad \xi = \frac{Da\cdot x\cdot Ra}{k_{r} } \left( \frac{L}{t} \right) ^{2} \end{aligned}$$

The dimensionless form of Eq. (37) can be written as

39 $$\frac{d^{2} \theta }{d\zeta ^{2} } -M^{2} \theta +M^{2} G(1+I_{G} \theta )-\xi \theta ^{2} =0$$

with its boundary condition

40 $$\left. \frac{d\theta }{d\zeta } \right| _{\zeta =0} =0, \quad and \quad \left. \theta \right| _{\zeta =1} =1.$$

where *M* is a convection parameter that indicates the effect of surface convecting of the fin and $\xi$ is a porous parameter that indicates the effect of the permeability of the porous medium as well as the buoyancy effect so higher the value of $\xi$ indicates higher permeability of the porous medium or higher buoyancy forces.

To understand the anomalous behaviour of this system, we fractionalize the energy balance Eq. (39) into fractional order ($\alpha >0$) in order to find the fin temperature in porous fins as,

41 $$\frac{d^{\alpha } \theta }{d\zeta ^{\alpha } } -M^{2} \theta + M^{2} G(1+I_{G} \theta )-\xi \theta ^{2} =0,\qquad 1<\alpha \le 2\; and\; 0\le \zeta \le 1$$

with boundary conditions

42 $$\left. \frac{d\theta }{d\zeta } \right| _{\zeta =0} =0 \quad and \quad \left. \theta \right| _{\zeta =1} =1.$$

Now, again applying sumudu transform on both sides of Eq. (41), it obtains

43 $$\begin{aligned} S\left[ \theta (\zeta )\right] = \theta _{0} (\zeta ) + u^{\alpha } S\left[ \left( M^{2} -M^{2} GI_{G} \right) \theta (\zeta )+\xi A_{n} (\theta ) - M^{2} G \right] \end{aligned}$$

Applying Inverse sumudu transfer on both sides, we get

44 $$\begin{aligned} \left[ \theta (\zeta )\right] = \theta _{0} (\zeta ) + S^{-1} \left[ u^{\alpha } S \left[ \left( M^{2} -M^{2} GI_{G} \right) \theta (\zeta ) +\xi A_{n} (\theta ) - M^{2} G\right] \right] \end{aligned}$$

by applying Adomian Decomposition Method, it obtains the following equation

45 $$\begin{aligned} \sum _{n=0}^{\infty }\lambda ^{n} \theta _{n} (\zeta )&= K - \lambda \left[ S^{-1} \left[ u^{\alpha } \left( M^{2}G \right) \right] \right] \\&\quad + S^{-1}\left[ u^{\alpha } S\left[ \sum _{n=0}^{\infty }\lambda ^{n} \left( \theta _{n} (\zeta )\left( M^{2} -M^{2} GI_{G} \right) +\sum _{n=0}^{\infty }\lambda ^{n} \xi A_{n} (\theta )\right) \right] \right] \end{aligned}$$

where $A_{n} (\theta )$ is the nonlinear term which can be determined by using Eq. (13). The first few components of the adomian’s polynomial for corrosponding nonlinear terms are given by

$$\begin{aligned} A_0&= {({\theta _0}(\zeta ))^2} \\ A_1&= 2{\theta _0}(\zeta ){\theta _1}(\zeta ) \\ A_2&= 2{\theta _0}(\zeta ){\theta _2}(\zeta ) + {({\theta _1}(\zeta ))^2} \\ A_3&= 2{\theta _0}(\zeta ){\theta _3}(\zeta ) + 2{\theta _1}(\zeta ){\theta _2}(\zeta ) \\ \vdots&\;&\end{aligned}$$

On comparing the coefficients of like powers of $\lambda$ in Eq. (45), we get decomposition components as

46 $$\begin{aligned} \theta _{0} (\zeta )&= K \\ \theta _{1} (\zeta )&= S^{-1} \left[ u^{\alpha } S \left[ \left( M^{2} -M^{2} GI_{G} \right) \theta _{0} (\zeta ) +\xi A_{0} (\theta ) - M^{2} G\right] \right] \\ \theta _{n+1} (\zeta )&= S^{-1} \left[ u^{\alpha } S \left[ \left( M^{2} -M^{2} GI_{G} \right) \theta _{n} (\zeta ) +\xi A_{n} (\theta )\right] \right], \quad n>1. \end{aligned}$$

By solving above equations, we get

$$\begin{aligned} \theta _{{0}} \left( \zeta \right)&= K \\ \theta _{{1}} \left( \zeta \right)&= \frac{{\left( {\left( {{M^2} - {M^2}G{I_G}} \right) K + \xi {K^2} - {M^2}G} \right) {X^\alpha }}}{{\Gamma \left( {\alpha + 1} \right) }} \\ \theta _{{2}} \left( \zeta \right)&= \frac{{\left( {{M^2} - {M^2}G{I_G}} \right) \left( {\left( {{M^2} - {M^2}G{I_G}} \right) K + \xi {K^2} - {M^2}G} \right) {X^{2 \alpha }}}}{{\Gamma \left( {2 \alpha + 1} \right) }} \\&\quad + \frac{{2K\xi \left( {\left( {{M^2} - {M^2}G{I_G}} \right) K + \xi {K^2} - {M^2}G} \right) {X^{2 \alpha }}}}{{\Gamma \left( {2 \alpha + 1} \right) }} \\ \theta _{{3}} \left( \zeta \right)&= \frac{{{{\left( {{M^2} - {M^2}G{I_G}} \right) }^2}\left( {\left( {{M^2} - {M^2}G{I_G}} \right) K + \xi {K^2} - {M^2}G} \right) {X^{3 \alpha }}}}{{\Gamma \left( {3 \alpha + 1} \right) }} \\&\quad + \frac{{\xi {{\left( {\left( {{M^2} - {M^2}G{I_G}} \right) K + \xi {K^2} - {M^2}G} \right) }^2}\Gamma \left( {2 \alpha + 1} \right) {X^{3 \alpha }}}}{{{{\left( {\Gamma \left( {\alpha + 1} \right) } \right) }^2}\Gamma \left( {3 \alpha + 1} \right) }}\\&\quad + \frac{{4K\xi \left( {\left( {{M^2} - {M^2}G{I_G}} \right) K + \xi {K^2} - {M^2}G} \right) \left( {{M^2} - {M^2}G{I_G}} \right) {X^{3 \alpha }}}}{{\Gamma \left( {3 \alpha + 1} \right) }} \\&\quad + \frac{{4{K^2}{\xi ^2}\left( {\left( {{M^2} - {M^2}G{I_G}} \right) K + \xi {K^2} - {M^2}G} \right) {X^{3 \alpha }}}}{{\Gamma \left( {3 \alpha + 1} \right) }} \\ \vdots&\;&\end{aligned}$$

The approximate solution of $\theta \left( \zeta \right)$ up to four terms becomes

47 $$\begin{aligned} \theta \left( \zeta \right)&= K + \frac{{\left( {\left( {{M^2} - {M^2}G{I_G}} \right) K + \xi {K^2} - {M^2}G} \right) {X^\alpha }}}{{\Gamma \left( {\alpha + 1} \right) }} \\&\quad + \frac{{\left( {{M^2} - {M^2}G{I_G}} \right) \left( {\left( {{M^2} - {M^2}G{I_G}} \right) K + \xi {K^2} - {M^2}G} \right) {X^{2 \alpha }}}}{{\Gamma \left( {2 \alpha + 1} \right) }} \\&\quad + \frac{{2K\xi \left( {\left( {{M^2} - {M^2}G{I_G}} \right) K + \xi {K^2} - {M^2}G} \right) {X^{2 \alpha }}}}{{\Gamma \left( {2 \alpha + 1} \right) }} \\&\quad + \frac{{{{\left( {{M^2} - {M^2}G{I_G}} \right) }^2}\left( {\left( {{M^2} - {M^2}G{I_G}} \right) K + \xi {K^2} - {M^2}G} \right) {X^{3 \alpha }}}}{{\Gamma \left( {3 \alpha + 1} \right) }} \\&\quad + \frac{{\xi {{\left( {\left( {{M^2} - {M^2}G{I_G}} \right) K + \xi {K^2} - {M^2}G} \right) }^2}\Gamma \left( {2 \alpha + 1} \right) {X^{3 \alpha }}}}{{{{\left( {\Gamma \left( {\alpha + 1} \right) } \right) }^2}\Gamma \left( {3 \alpha + 1} \right) }} \\&\quad + \frac{{4K\xi \left( {\left( {{M^2} - {M^2}G{I_G}} \right) K + \xi {K^2} - {M^2}G} \right) \left( {{M^2} - {M^2}G{I_G}} \right) {X^{3 \alpha }}}}{{\Gamma \left( {3 \alpha + 1} \right) }} \\&\quad + \frac{{4{K^2}{\xi ^2}\left( {\left( {{M^2} - {M^2}G{I_G}} \right) K + \xi {K^2} - {M^2}G} \right) {X^{3 \alpha }}}}{{\Gamma \left( {3 \alpha + 1} \right) }} \end{aligned}$$

where value of *K* can be determined at the boundary condition $\left. \theta \right| _{\zeta =1} =1$ using Eq. (47). Since a constant *K* is assumed as an initial guess, it automatically satisfies the given boundary condition.

## Stability analysis via fixed point theorem

Let $\left( {X,\left\| \cdot \right\| } \right)$ be a Banach space and *H* a self-map of *X*. Let $\theta _{n+1} = f(H,\theta _{n})$ be particular recursive procedure. Suppose that *F*(*H*) the fixed point set of *H* has at lease one element and that $\theta _{n}$ converges to a point $p \in F(H)$. Let ${\theta _{n}\subseteq X}$ and define ${e_n} = \left\| {{\theta _{n + 1}} - f(H,{\theta _n})} \right\|$. If $\mathop {\lim }\limits _{n \rightarrow \infty } {\theta ^n} = p$, then the iteration $\theta _{n+1}=f(H,\theta _{n})$ is said to be *H*-stable. Without any loss of generality, we must assume that, our sequence ${\theta _{n}}$ has an upper boundary; otherwise we cannot expect the possibility of convergence. If all these conditions are satisfied for $\theta _{n+1}=H \theta _{n}$ which is known as Picard’s iteration, then the iteration will be *H*-Stable. Now we state the following theorem (Atangana 2016).

### **Theorem 1**

*Let*$\left( {X,\left\| \right\| } \right)$*be a Banach space andHa self-map ofXsatisfying*

48 $$\left\| {{H_x} - {H_y}} \right\| \le C\left\| {x - {H_x}} \right\| + c\left\| {x - y} \right\| ,$$

*for allx*, *yinXwhere*$0 \le C.\;0 \le c < 1.$*ThenHis Picard H-Stable*.

Let the following succession correlate to the nonlinear fractional order energy balance equation (Eq. 46),

$$\begin{aligned} \theta _{n+1} (\zeta ) = S^{-1} \left[ u^{\alpha } S\left[ \theta _{n} (\zeta )\left( M^{2} -M^{2} GI_{G} \right) +\xi (A_{n} (\theta ))\right] \right] ,\quad n>1. \end{aligned}$$

### **Theorem**

*LetTbe a self-map defined as*

49 $$\begin{aligned} T(\theta _{n}(\zeta ))&= \theta _{n+1} (\zeta ) \\&= S^{-1} \left[ u^{\alpha } S\left[ \theta _{n} (\zeta )\left( M^{2} -M^{2} GI_{G} \right) +\xi (A_{n} (\theta ))\right] \right] \end{aligned}$$

*is T-stable in*$L^{2}(a, b)$*if*$\left\{ \beta _{1}+\beta _{1}\kappa \right\} < 1.$

### *Proof*

The first step of the proof will consist on showing that *T* has a fixed point. To achieve this, we evaluate the following for all $(n,k)\in N \times N$

50 $$\begin{aligned} \left\| {T({\theta _n}(\zeta )) - T({\theta _k}(\zeta ))} \right\| = \left\| \begin{array}{l} {S^{ - 1}}\left[ {{u^\alpha }S\left[ {\left( {{M^2} - {M^2}G{I_G}} \right) {\theta _n}(\zeta ) + \xi (\theta _n^2(\zeta ))} \right] } \right] \\ - {S^{ - 1}}\left[ {{u^\alpha }S\left[ {\left( {{M^2} - {M^2}G{I_G}} \right) {\theta _k}(\zeta ) + \xi (\theta _k^2(\zeta ))} \right] } \right] \end{array} \right\| \end{aligned}$$

Using the linearity property of the inverse sumudu transform, we obtain

51 $$\begin{aligned} \left\| {T({\theta _n}(\zeta )) - T({\theta _k}(\zeta ))} \right\| = \left\| {{S^{ - 1}}\left[ {{u^\alpha }S\left\{ \begin{array}{l} \left( {{M^2} - {M^2}G{I_G}} \right) \left( {{\theta _n}(\zeta ) - {\theta _k}(\zeta )} \right) \\ + \xi (\theta _n^2(\zeta ) - \theta _k^2(\zeta )) \end{array} \right\} } \right] } \right\| \end{aligned}$$

Using the triangle inequality for the norm, we get

52 $$\begin{aligned} \left\| {T({\theta _n}(\zeta )) - T({\theta _k}(\zeta ))} \right\|&\le \left\| {{S^{ - 1}}\left[ {{u^\alpha }S\left\{ {\left( {{M^2} - {M^2}G{I_G}} \right) \left( {{\theta _n}(\zeta ) - {\theta _k}(\zeta )} \right) } \right\} } \right] } \right\| \\&\quad + \left\| {{S^{ - 1}}\left[ {{u^\alpha }S\left\{ {\xi (\theta _n^2(\zeta ) - \theta _k^2(\zeta ))} \right\} } \right] } \right\| \end{aligned}$$

The above can be further be transformed using the property of norm and integral as follows

53 $$\begin{aligned} \left\| {T({\theta _n}(\zeta )) - T({\theta _k}(\zeta ))} \right\|&\le {S^{ - 1}}\left[ {{u^\alpha }S\left\{ {\left\| {\left\{ {\left( {{M^2} - {M^2}G{I_G}} \right) \left( {{\theta _n}(\zeta ) - {\theta _k}(\zeta )} \right) } \right\} } \right\| } \right\} } \right] \\&\quad + {S^{ - 1}}\left[ {{u^\alpha }S\left\{ {\left\| {\left\{ {\xi (\theta _n^2(\zeta ) - \theta _k^2(\zeta ))} \right\} } \right\| } \right\} } \right] \end{aligned}$$

The evaluation of Eq. (53) can be done as follows

54 $$\begin{aligned} \left\| {\left( {{M^2} - {M^2}G{I_G}} \right) \left\{ {{\theta _n}(\zeta ) - {\theta _k}(\zeta )} \right\} } \right\|&\le \left\| {\left( {{M^2} - {M^2}G{I_G}} \right) } \right\| \left\| {{\theta _n}(\zeta ) - {\theta _k}(\zeta )} \right\| \\& \le {\beta _1}\left\| {{\theta _n}(\zeta ) - {\theta _k}(\zeta )} \right\| \end{aligned}$$

and

55 $$\begin{aligned} \left\| {\xi \left\{ {\theta _n^2(\zeta ) - \theta _k^2(\zeta )} \right\} } \right\|& \le \left\| \xi \right\| \left\| {\theta _n^2(\zeta ) - \theta _k^2(\zeta )} \right\| \\ & \le {\beta _2}\left\| {{\theta _n}(\zeta ) + {\theta _k}(\zeta )} \right\| \left\| {{\theta _n}(\zeta ) - {\theta _k}(\zeta )} \right\| \\& \le {\beta _2}\kappa \left\| {{\theta _n}(\zeta ) - {\theta _k}(\zeta )} \right\| \end{aligned}$$

where $\left\| \xi \right\| \beta _2.$ Now putting together Eqs. (54) and (55) into Eq. (53), we obtain the following

56 $$\begin{aligned} \left\| {T({\theta _n}(\zeta )) - T({\theta _k}(\zeta ))} \right\| \le \left\{ {{\beta _1} + {\beta _2}k} \right\} \left\| {{\theta _n}(\zeta ) - {\theta _k}(\zeta )} \right\| \end{aligned}$$

with $\left\{ {{\beta _1} + {\beta _2}\kappa } \right\} < 1, \forall \beta _1 \beta _2.$ Hence, the nonlinear T-self mapping has a fixed point. This completes the proof. Further we show that, *T* satisfies the condition in Theorem 1, Now for

57 $$C = 0, \,\, c = \left\{ {{\beta _1} + {\beta _2}k} \right\}$$

shows that conditions of this theorem holds for the nonlinear mapping *T*. Since all condition in Theorem 1 hold for the defined non-linear mapping *T*. Hence, *T* is Picard’s *T*-stable. This completes the proof of this theorem. $\square$

## Results and discussion

The purpose of this work is to observe the simultaneous effects of the governing parameters and the different fractional values $\alpha$ on solid and porous fins. The range of thermal and physical parameters selected the present work is shown in Table 1.

**Table 1:** Range of values for physical and thermal parameters

Parameters	*M*	*G*	$I_{G}$	$\xi$
Values	0.5, 1 and 5	0.1–0.9	0.1–0.9	0.1–0.9

### Solid fin with temperature dependent internal heat generation and constant thermal conductivity

**Fig. 2:**
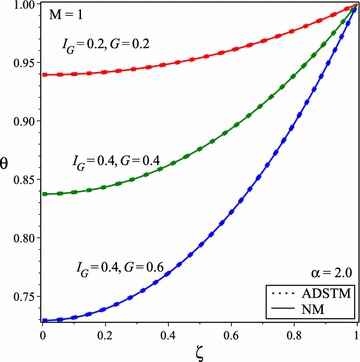
Temperature variation obtained by ADSTM in comparison with the numerical solution for $\alpha = 2$ and *M* = 1

**Fig. 3:**
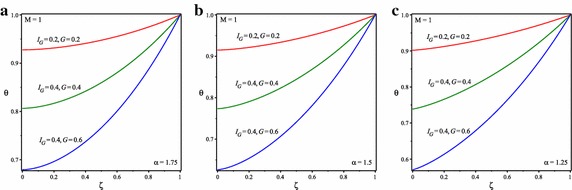
Temperature distribution in solid fins for *M* = 1 and **a** $\alpha = 1.75$, **b** $\alpha =1.5$, **c** $\alpha =1.25$

Temperature distribution for this case (temperature dependent heat generation and constant thermal conductivity) is shown in Figs. 2 and 3 where M = 1 that is common in fin design.

Figure 2 shows the temperature distribution for this state and $I_{G} =G=0.2$, $I_{G} =G=0.4$ and $I_{G} =0.4$, *G* = 0.6. This choice of parameters represents a fin with moderate temperature dependent heat generation and the thermal conductivity variation of 20 % between the base and the surrounding coolant temperatures that are often used in nuclear rods.

It is shown in Figs. 2 and 3 that temperature of the fin increases by increasing the value of $I_{G}$ and *G* because of increasing in heat generation. The comparison of obtained results with numerical results reveals that ADSTM has good efficiency and accuracy.

Figure 3 shows the temperature distribution for this state and $I_{G} =G=0.2, I_{G} =G=0.4$ and $I_{G} =0.4$, *G* = 0.6 and for the different fractional order value of $\alpha$ = 1.75, 1.5 and 1.25. Further, the nature of the graphs depicts that, considered value of $\alpha$ represents the point of convergence under the given range of interval between 1 and 2.

### Porous fin with temperature dependent internal heat generation

**Fig. 4:**
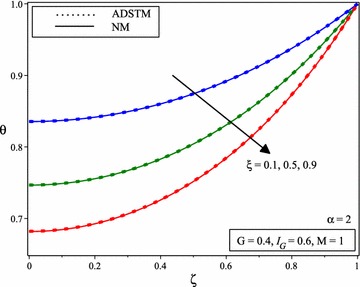
Comparison of dimensionless temperature distribution in porous fins with temperature dependent international heat generation for the different values of porous parameter and $\alpha =2$

**Fig. 5:**
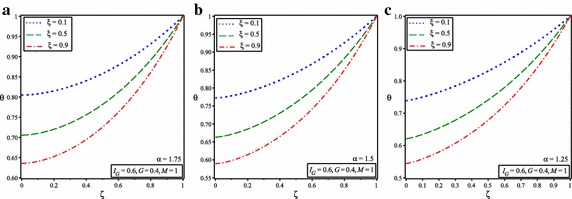
Temperature distribution of porous fin with temperature dependent internal heat generation for the different values of porous parameter and for **a** $\alpha =1.75$, **b** $\alpha =1.5$, **c** $\alpha =1.25$

Figure 4 shows the comparison between the numerical solution and the ADSTM solution for temperature distribution when *M* = 1, *G* = 0.4, $I_{G} =0.6$ and for the different value of $\xi$ and $\alpha =2$. When the porous parameter $\xi$ increases, it can be noticed from Fig. 5 that, a declines in fin temperature which causes stronger cooling results decrease in temperature distribution. Figure 5 shows the variation of temperature distribution of porous fin with temperature dependent internal heat generation when *M* = 1, *G* = 0.4, $I_{G} =0.6$ and for the different value of $\xi$ and for $\alpha$ = 1.75, 1.5, 1.25.

**Fig. 6:**
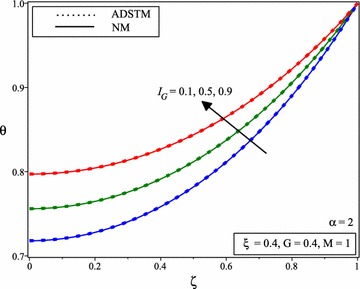
Comparison of dimensionless temperature distribution in porous fins with temperature dependent international heat generation for the different values of internal heat generation parameter and $\alpha =2$

**Fig. 7:**
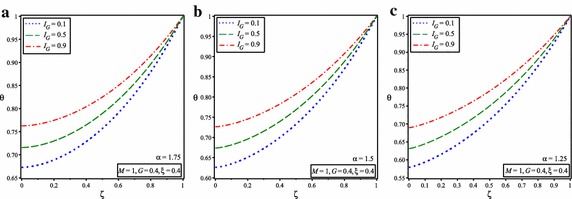
Temperature distribution of porous fin with temperature dependent internal heat generation for the different values of internal heat generation parameter and for **a** $\alpha =1.75$, **b** $\alpha =1.5$, **c** $\alpha =1.25$

The variation of the temperature distribution along the fin for the different values of internal heat generation, $I_{G}$ when *M* = 1, *G* = 0.4, $\zeta =0.4$ and for $\alpha =2$ is illustrated in Fig. 6. It is observed from figure that, if the internal heat generation increases then fin temperature be increases. Figure 7 shows the variation of temperature distribution of porous fin with temperature dependent internal heat generation when *M* = 1, *G* = 0.4, $\xi =0.4$ and for the different value of $I_{G}$ and for $\alpha =1.75, 1.5, 1.25$.

**Fig. 8:**
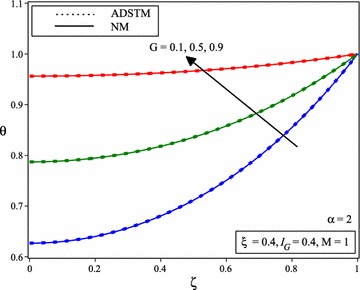
Comparison of dimensionless temperature distribution in porous fins with temperature dependent international heat generation for the different values of heat generation parameter and $\alpha =2$

Figure 8 shows the comparison between the numerical solution and the ADSTM solution for temperature distribution when *M* = 1, $I_{G} =0.4, \xi =0.4$ and for the different value of *G* and for $\alpha =2$.

**Fig. 9:**
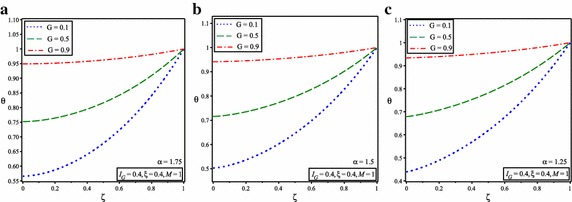
Temperature distribution of porous fin with temperature dependent internal heat generation for the different values of heat generation parameter and for **a** $\alpha =1.75$, **b** $\alpha =1.5$, **c** $\alpha =1.25$

Figure 9 shows the variation of temperature distribution of porous fin with temperature dependent internal heat generation when *M* = 1, $I_{G} =0.4, \xi =0.4$ and for the different value of *G* and for $\alpha =1.75, 1.5, 1.25$.

**Fig. 10:**
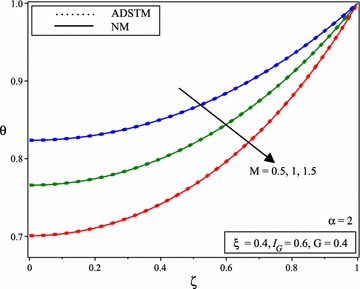
Comparison of dimensionless temperature distribution in porous fins with temperature dependent international heat generation for the different values of convective parameter and $\alpha =2$

**Fig. 11:**
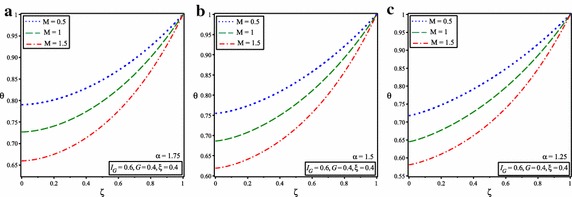
Temperature distribution of porous fin with temperature dependent internal heat generation for the different values of convective parameter and for **a** $\alpha =1.75$, **b** $\alpha =1.5$, **c** $\alpha =1.25$

Figure 10 shows the comparison between the numerical solution and ADSTM solution for temperature distribution when *G* = 0.4, $I_{G} =0.6, \xi =0.4$ and for the different value of *M* and for $\alpha =2$. At last, it can be concluded that the analytical results correspond exactly with the numerical results. This means that the ADSTM has a high aptitude in solving highly nonlinear initial and boundary value problems without involving linearization. Figure 11 shows the variation of temperature distribution of porous fin with temperature dependent internal heat generation when *G* = 0.4, $I_{G} =0.6, \xi =0.4$ and for the different value of *M* and for $\alpha =1.75, 1.5, 1.25$.

## Conclusion

In this study, the heat transfer in rectangular solid and porous fin with the temperature dependent internal heat generation is analyzed by using ADSTM and used the concept of *T*-stable mapping and the fixed point theorem to prove the stability of ADSTM. Here, it is shown that, ADSTM provide a simple, accurate and appropriate technique for simulating the heat transfer in solid and porous fin in a fractional order energy balance equation. The results shows that the temperature distribution strongly depends on different parameter in solid fin as well as on Darcy’s number in porous fin and also on the fractional parameter.

## References

[CR1] Adomian G (1994). Solving frontier problems of physics: the decomposition method.

[CR2] Alkam M, Al-Nimr M, Hamdan M (2002). On forced convection in channels partially filled with porous substrates. Heat Mass Transf.

[CR3] Alkam M, Al-Nimr M (1999). Solar collectors with tubes partially filled with porous substrates. J Sol Energy Eng.

[CR4] Atangana A (2016). On the new fractional derivative and application to nonlinear fisher’s reaction–diffusion equation. Appl Math Comput.

[CR5] Atangana A, Alqahtani RT (2016). Modelling the spread of river blindness disease via the caputo fractional derivative and the beta-derivative. Entropy.

[CR6] Atangana A, Baleanu D (2013) Nonlinear fractional Jaulent–Miodek and Whitham–Broer–Kaup equations within sumudu transform. In: Abstract and applied analysis, vol 2013. Hindawi Publishing Corporation

[CR7] Atangana A, Baleanu D (2016) New fractional derivatives with nonlocal and non-singular kernel: theory and application to heat transfer model. arXiv preprint arXiv:1602.03408

[CR8] Baskonus HM, Bulut H (2015). On the numerical solutions of some fractional ordinary differential equations by fractional Adams–Bashforth–Moulton method. Open Math.

[CR9] Baskonus HM, Bulut H (2015). Analytical studies on the (1 + 1)-dimensional nonlinear dispersive modified Benjamin–Bona–Mahony equation defined by seismic sea waves. Waves Random Complex Media.

[CR10] Belgacem FBM, Karaballi AA (2006). Sumudu transform fundamental properties investigations and applications. Int J Stoch Anal.

[CR11] Bergman TL, Incropera FP, Lavine AS, Dewitt DP (2011). Fundamentals of heat and mass transfer.

[CR12] Ghasemi S, Valipour P, Hatami M, Ganji D (2014). Heat transfer study on solid and porous convective fins with temperature-dependent heat generation using efficient analytical method. J Cent South Univ.

[CR13] Gorla RSR, Bakier A (2011). Thermal analysis of natural convection and radiation in porous fins. Int Commun Heat Mass Transf.

[CR14] Hamdan M, Al-Nimr M, Alkam M (2000). Enhancing forced convection by inserting porous substrate in the core of a parallel-plate channel. Int J Numer Methods Heat Fluid Flow.

[CR15] Hamdan M, Moh’d AA-N (2010). The use of porous fins for heat transfer augmentation in parallel-plate channels. Transp Porous Media.

[CR16] Hatami M, Hasanpour A, Ganji D (2013). Heat transfer study through porous fins (si 3 n 4 and al) with temperature-dependent heat generation. Energy Convers Manag.

[CR17] Hatami M, Ganji D (2013). Thermal performance of circular convective–radiative porous fins with different section shapes and materials. Energy Convers Manag.

[CR18] Jarad F, Bayram K, Abdeljawad T, Baleanu D (2012). On the discrete sumudu transform. Rom Rep Phys.

[CR19] Kadem A, Baleanu D (2012). Two-dimensional transport equation as fredholm integral equation. Commun Nonlinear Sci Numer Simul.

[CR20] Kiwan S (2007). Effect of radiative losses on the heat transfer from porous fins. Int J Therm Sci.

[CR21] Kiwan S (2007). Thermal analysis of natural convection porous fins. Transp Porous Media.

[CR22] Kiwan S, Al-Nimr M (2001). Using porous fins for heat transfer enhancement. Tc.

[CR23] Kiwan S, Zeitoun O (2008). Natural convection in a horizontal cylindrical annulus using porous fins. Int J Numer Methods Heat Fluid Flow.

[CR24] Miller KS, Ross B (1993). An introduction to the fractional calculus and fractional differential equations.

[CR25] Nield DA, Bejan A (2006). Convection in porous media.

[CR26] Patel T, Meher R (2015). A study on temperature distribution, efficiency and effectiveness of longitudinal porous fins by using adomian decomposition sumudu transform method. Procedia Eng.

[CR27] Patel T, Meher R (2015). Adomian decomposition sumudu transform method for solving fully nonlinear fractional order power-law fin-type problems. Int J Math Comput.

[CR28] Roshid HO, Kabir MR, Bhowmik RC, Datta BK (2014). Investigation of solitary wave solutions for Vakhnenko–Parkes equation via exp-function and exp (- $$\phi $$ ($$\xi $$))-expansion method. SpringerPlus.

[CR29] Roshid HO, Hoque MF, Akbar MA (2014). New extended ($$g^{\prime }/g$$)-expansion method for traveling wave solutions of nonlinear partial differential equations (npdes) in mathematical physics. Ital J Pure Appl Math.

[CR30] Srivastava H, Golmankhaneh AK, Baleanu D, Yang X-J (2014) Local fractional sumudu transform with application to IVPs on cantor sets. In: Abstract and applied analysis, vol 2014. Hindawi Publishing Corporation

[CR31] Watugala G (1993). Sumudu transform: a new integral transform to solve differential equations and control engineering problems. Integr Educ.

